# *HLA-G* 3′UTR Polymorphisms Predict Drug-Induced G3-4 Toxicity Related to Folinic Acid/5-Fluorouracil/Oxaliplatin (FOLFOX4) Chemotherapy in Non-Metastatic Colorectal Cancer

**DOI:** 10.3390/ijms18071366

**Published:** 2017-06-27

**Authors:** Marica Garziera, Saverio Virdone, Elena De Mattia, Lucia Scarabel, Erika Cecchin, Jerry Polesel, Mario D’Andrea, Nicoletta Pella, Angela Buonadonna, Adolfo Favaretto, Giuseppe Toffoli

**Affiliations:** 1 Experimental and Clinical Pharmacology Unit, CRO Aviano National Cancer Institute, IRCCS, via F. Gallini 2, Aviano (PN) 33081, Italy; edemattia@cro.it (E.D.M.); lucia.scarabel@cro.it (L.S.); ececchin@cro.it (E.C.); gtoffoli@cro.it (G.T.); 2 Unit of Cancer Epidemiology, CRO Aviano National Cancer Institute, IRCCS, via F. Gallini 2, Aviano (PN) 33081, Italy; svirdone@cro.it (S.V.); polesel@cro.it (J.P.); 3 Medical Oncology Unit, “San Filippo Neri Hospital”, Piazza Di S. Maria Della Pietà, Rome 00135, Italy; mariorosario.dandrea@aslroma1.it; 4 Medical Oncology Unit, University Hospital, Piazzale Santa Maria della Misericordia 15, Udine (UD) 33100, Italy; nicoletta.pella@asuiud.sanita.fvg.it; 5 Medical Oncology Unit B, CRO Aviano National Cancer Institute, IRCCS, via F. Gallini 2, Aviano (PN) 33081, Italy; abuonadonna@cro.it; 6 Medical Oncology Unit, Ospedale Cà Foncello, Piazzale Ospedale 1, Treviso (TV) 31100, Italy; adolfo.favaretto@aulss2.veneto.it

**Keywords:** colorectal cancer, *human leukocyte antigen-G* 3′UTR, polymorphism, toxicity, folinic acid/5-fluorouracil/oxaliplatin, immunogenetics

## Abstract

Polymorphisms in drug-metabolizing enzymes might not completely explain inter-individual differences in toxicity profiles of patients with colorectal cancer (CRC) that receive folinic acid/5-fluorouracil/oxaliplatin (FOLFOX4). Recent data indicate that the immune system could contribute to FOLFOX4 outcomes. In light of the immune inhibitory nature of human leukocyte antigen-G (HLA-G), a non-classical major histocompatibility complex (MHC) class I molecule, we aimed to identify novel genomic markers of grades 3 and 4 (G3-4) toxicity related to FOLFOX4 therapy in patients with CRC. We retrospectively analyzed data for 144 patients with stages II-III CRC to identify *HLA-G* 3′ untranslated region (3′UTR) polymorphisms and related haplotypes and evaluate their impact on the risk of developing G3-4 toxicities (i.e., neutropenia, hematological/non-hematological toxicity, neurotoxicity) with logistic regression. The rs1610696-*G/G* polymorphism was associated with increased risk of G3-4 neutropenia (OR = 3.76, *p* = 0.015) and neurotoxicity (OR = 8.78, *p* = 0.016); rs371194629-*Ins/Ins* was associated with increased risk of neurotoxicity (OR = 5.49, *p* = 0.027). *HLA-G* 3′UTR-2, which contains rs1610696-*G/G* and rs371194629-*Ins/Ins* polymorphisms, was associated with increased risk of G3-4 neutropenia (OR = 3.92, *p* = 0.017) and neurotoxicity (OR = 11.29, *p* = 0.009). A bootstrap analysis confirmed the predictive value of rs1610696 and rs371194629, but the UTR-2 haplotype was validated only for neurotoxicity. This exploratory study identified new *HLA-G* 3′UTR polymorphisms/haplotypes as potential predictive markers of G3-4 toxicities in CRC.

## 1. Introduction

Colorectal cancer (CRC) is the third most frequent cancer and the second leading cause of cancer death in the world, in both women and men [1,2]. The management of patients with CRC is very complex [3,4,5]; therapeutic decisions are generally based on the tumor stage established at the time of diagnosis [6,7]. Despite the high incidence of this neoplasia, the majority of patients newly diagnosed with CRC have a localized early stage disease that is suitable for curative surgical resection [8]. In stage III colon cancer that presents with ganglion involvement, administration of adjuvant chemotherapy (CT) is recommended, in addition to surgical intervention to reduce the likelihood of relapse and increase the chances of cure. The use of adjuvant treatment in stage II CRC is more controversial, due to the lower risk of recurrence [9]; thus, adjuvant CT is considered an acceptable treatment option in stage II CRC only for patients at high-risk [3]. Standard adjuvant regimens include oxaliplatin (OXA) combined with folinic acid/5-fluorouracil (5-FU) or capecitabine (XELOX) [3,8]. Compared to the use of fluoropyrimidines (FLs; i.e., 5-FU/folinic acid or capecitabine) as a single agent, the combination with the platinum derivate significantly increased response rates and improved survival chances [3,9,10]. On the other hand, the addition of OXA to FLs was burdened by a higher incidence of toxicity. Several patients experienced mild or moderate side effects at some point during treatment [11,12]. The toxicity profile of the FLs/OXA combination is well-documented; the most frequently reported serious (≥G3) adverse events of these regimens in Western populations are hematologic (neutropenia in 40–56% of patients) and gastrointestinal (GI) events (diarrhea in 10–15% of patients), linked to FLs administration, and peripheral sensory neuropathy [13,14], linked to acute and cumulative doses of OXA [12]. Neurotoxicity may be irreversible, and/or it may last long after cessation of CT in 10–20% of patients [12].

Pharmacogenomics has been largely applied in recent years to the personalization of adjuvant treatments for CRC [15,16]. Several candidate-gene investigations [17,18,19] and more recently, genome-wide association studies, have demonstrated that functional germline polymorphisms involved in the OXA and FLs pathways could contribute to inter-individual differences in the pharmacokinetics and toxicity profiles of patients [20]. In particular, to explain the variability in individual toxicity profiles, research efforts have focused on folate pathway markers (i.e., *DYPD*, *TYMS*, *MTHFR*) related to FLs toxicity; and they have focused on oxidative pathways (i.e., *GSTs*) and the DNA repair enzyme system (i.e., *ERCC1-2*, *XRCC1*, *MSH*) related to OXA toxicity [21,22,23,24]. Our group had also generated important results, which identified predictive markers of G3-4 toxicity, including variants in genes that encoded membrane transporters (*MRP1*, *MRP2*) and DNA repair enzymes (*MHS6*, *XRCC3*), in the same cohort evaluated here [25]. However, it was difficult to draw definitive conclusions based on currently available studies, due to the significant methodological drawbacks linked to study designs (i.e., retrospective analyses, small sample sizes, lack of data validation) and the high population heterogeneity [21]. Despite the great efforts made to identify genetic determinants of G3-4 toxicity in FLs/OXA-based therapy, we remain far from realizing optimized treatments, based on a set of validated genetic biomarkers [15]. This shortcoming probably reflects insufficient knowledge of the biological mechanisms underlying the toxicity linked to FLs and OXA, and it suggests the involvement of pathways other than those investigated to date.

Recent preliminary evidence has indicated that the antitumor effects of platinum derivates partly arise from modulation of the immune system. These immunogenic effects include, among others, modulation of signal transducer and activator of transcription (STAT) signaling and enhancement of immune effector responses [26,27]. Immune processes have also been reported to play a key role in the pathogenesis of FLs-related GI toxicity. In particular, inflammatory mediators, such as tumor necrosis factor- α (TNF-α) and interleukin-1β (IL-1β), were implicated in the complex mechanism underlying the toxic damage to GI epithelium (i.e., mucositis) induced by some CTs, including 5-FU [28]. The *human leukocyte antigen-G* (*HLA-G*) locus is a non-classic major histocompatibility complex (MHC) class I gene which exhibits well recognized immune-inhibitory effects on both innate and adaptive immune responses [29,30,31]. HLA-G is highly expressed in immune privileged sites, such as trophoblasts during placentation, and its distribution is restricted in normal tissues [32]. Nevertheless, HLA-G participates in the tumor escape phenomenon [31,33] because its expression can be induced in pathological conditions, like cancer [34]. HLA-G overexpression has been observed in primary CRC lesions [35,36] and it was associated with reduced overall survival [37,38]. In fact, soluble HLA-G is considered a marker for CRC [39,40]. Genetic variations in the untranslated regions (UTRs) of *HLA-G*, particularly in the 3′UTR, were shown to be involved in *HLA-G* regulation [41,42]. In this non-coding region, single nucleotide polymorphisms (SNPs) play a prominent role in influencing *HLA-G* RNA turnover, stability, splicing [43,44], and therefore, HLA-G protein expression [45]. Some 3′UTR SNPs of the *HLA-G* gene were shown to be independently associated with cancer susceptibility [46,47,48], and recently, with CRC prognosis [49]. However, no data have been reported to date regarding the role of *HLA-G* variants in the modulation of CT-related toxicity in CRC and in other tumor settings. The present study aimed to evaluate a panel of 8 *HLA-G* 3′UTR polymorphisms and determine their effects on the risk of G3-4 cumulative toxicity related to adjuvant folinic acid/5-fluorouracil/oxaliplatin-based (FOLFOX4-based) therapy in 144 patients with non-metastatic CRC. Our goal was to define novel genetic markers that may improve differential predictions of the risk of toxicity.

## 2. Results

### 2.1. Patient Characteristics

The main demographic and clinical characteristics of eligible patients with CRC (*n* = 144) are shown in Table 1.

The majority of tumors were in stage III at the time of diagnosis (*n* = 123, 85.4%), and the most common primary tumor site was the colon (*n* = 111, 77.1%). Adjuvant CT was homogenously administered as 5-FU plus OXA, according to the FOLFOX4 regimen, in all patients [25]. Out of 33 patients with primary rectal cancer, 19 (57.6%) received neo-adjuvant radiotherapy. About half of the patients (*n* = 73; 50.7%) experienced at least one G3-4 adverse event (Table 2); the most common (*n* = 53, 36.8%) hematological adverse event was G3-4 neutropenia; the most frequent non-hematological G3-4 toxicities were diarrhea (*n* = 16, 11.1%) and vomiting (*n* = 5, 3.5%).

### 2.2. Germline HLA-G 3′UTR Polymorphisms and Haplotypes

Nine germline SNPs in the *HLA-G* 3′UTR that were previously genotyped [49] were considered in this study. Only 8 polymorphisms had ≥5% variant allelic frequency (rs371194629, rs1707, rs1710, rs17179101, rs17179108, rs1063320, rs9380142, and rs1610696) and were included in the analysis. All selected SNPs exhibited genotype distributions that conformed to the assumptions of the Hardy-Weinberg equilibrium. We observed strong linkage disequilibrium between +3010 C>G and +3142 G>C polymorphisms (not shown), as previously reported [49]. Finally, the PHASE method was employed to reconstruct the *HLA-G* 3′UTR haplotypes from the unphased gametic genotype data at the eight variation sites for each of the 144 patients with CRC. A total of 12 different haplotypes (Table S1) were defined, and all were previously described [49,50]. The most highly represented haplotypes were (in descending order): UTR-2 (103/288, 35.8%), UTR-1 (74/288, 25.7%), UTR-3 (41/288, 14.2%), UTR-4 (27/288, 9.4%), UTR-7 (17/288, 5.9%), UTR-5 (11/288, 3.8%), and UTR-6 (9/288, 3.1%). These seven haplotypes represented more than 97% of the total, but UTRs 1–4 accounted for 84.4%. Only haplotypes with frequencies >1% were included in the statistical analysis.

### 2.3. New Discoveries of Germline HLA-G 3UTR SNPs and G3-4 Toxicities

Our exploratory analysis with adjusted logistic regression models found significant associations between specific *HLA-G* 3UTR SNPs and G3-4 hematological toxicity, neutropenia, and neurotoxicity. In particular, rs1707 SNP was associated with reduced risk of G3-4 hematological toxicity (OR = 0.35, 95% CI: 0.12–0.98, *p* = 0.046), according to the dominant model (Table 3), and rs1610696 SNP was associated with increased risk of hematological toxicity (OR = 3.34, 95% CI: 1.16–9.59, *p* = 0.025), according to the recessive model. Only rs1610696 SNP was predictive of G3-4 neutropenia (recessive, OR = 3.76, 95% CI: 1.29–10.96, *p* = 0.015) (Table 4). We found that G3-4 neurotoxicity was associated with rs371194629 (OR = 5.49, 95% CI: 1.21–24.85, *p* = 0.027) and rs1610696 (OR = 8.78, 95% CI: 1.50–51.44, *p* = 0.016) polymorphisms (Table 5), both in the recessive model. None of the polymorphisms and/or haplotypes investigated was predictive of G3-4 non-hematological toxicities. Of note, significant associations with ≥G2 non-hematological, hematological, neutropenia, or neurological toxicities were not found. Bootstrap internal re-sampling confirmed the significant associations between rs1610696 SNP and three G3-4 toxicities: hematological (*p* = 0.038, Table 3), neutropenia (*p* = 0.042, Table 4), and neurotoxicity (*p* = 0.019, Table 5); also, rs371194629 SNP remained associated with neurotoxicity (*p* = 0.022, Table 5).

### 2.4. Associations between HLA-G 3′UTR Haplotypes and G3-4 Toxicities

Haplotypes of the *HLA-G* 3′UTR were also investigated to define the possible combinatorial effects of *HLA-G* 3′UTR polymorphisms on the risk of developing G3-4 toxicities with adjusted logistic regression models (Table 6). The UTR-2 (InsTCCCGAG) haplotype was associated with increased risk of hematological toxicity (recessive, OR = 3.46, 95% CI: 1.14–11.52, *p* = 0.028), and the UTR-4 (DelCGCCCAC) haplotype was associated with reduced risk of hematological toxicity (dominant, OR = 0.35, 95% CI: 0.12–0.98, *p* = 0.046). G3-4 neutropenia was associated with UTR-2 (recessive, OR = 3.92, 95% CI: 1.28–12.07, *p* = 0.017) and UTR-6 (DelTGCCCAC) (dominant, OR = 4.77, 95% CI: 1.07–21.20, *p* = 0.040) haplotypes. Finally, G3-4 neurotoxicity was associated with the UTR-2 (recessive, OR = 11.29, 95% CI: 1.84–69.40, *p* = 0.009) haplotype. Among these haplotypes, only the association between UTR-2 and G3-4 neurotoxicity was internally validated (*p* = 0.019, Table 6).

## 3. Discussion

Fluoropyrimidines and platinum-based CTs are greatly complicated by their associated side effects. In the last few decades, several polymorphic genetic variants were intensely investigated in different tumor settings. Of those, some were identified as potential predictors of toxicity, but the data were not robust, particularly data related to OXA-toxicity [20,22]. Hence, we reasoned that other, under-explored elements might significantly contribute to the likelihood of developing severe complications after adjuvant CT.

In light of the immune inhibitory nature of the HLA-G molecule [33,51], we aimed to find novel genomic markers of G3-4 toxicity among polymorphisms and related haplotypes in the 3′UTR of the *HLA-G* gene. We studied a set of 144 patients with non-metastatic (stage II-III) CRC, after curative resections and FOLFOX-based CT. To the best of our knowledge, this study was the first to explore the potential of *HLA-G* 3′UTR SNPs in predicting the risk of CT-related toxicities. Our present data represent the first evidence that rs1610696 and rs371194629 markers and the related UTR-2 haplotype could predict the likelihood of experiencing a G3-4 toxicity. No previous studies have described the role of these variants as predictors of severe adverse effects in CRC as well as in other cancer types. Anyway, some studies have described the impact of the same variants on tumor recurrence, overall survival and cancer risk. In particular, focusing on CRC setting, patients with non-metastatic CRC that carried the rs371194629-14-bp *Ins* allele exhibited prolonged disease-free survival after FLs/OXA based CT [49] and reduced CRC susceptibility [46]. In addition, considering other tumor types, some studies reported a significant correlation between the rs371194629-*Del/Del* genotype and reduced overall survival in patients with chronic lymphocytic leukemia [52] and diffuse large B-cell lymphoma after immunochemotherapy [53]. The rs1610696 SNP did not show any value as a prognostic marker [49], but similar to rs371194629, it was linked to increased CRC risk, particularly the rs1610696-*G/G* form, in the context of homozygous UTR-2 [46]. In vitro functional data and in vivo phenotypic evidence are available only for rs371194629 SNP; specifically, the presence of the rs371194629-+14-bp *Ins* allele was demonstrated to be associated with unstable *HLA-G* mRNAs, and therefore, lower levels of the protein [43]. The effects of the other significantly predictive markers remain to be elucidated. Considering the few functional and phenotypic data available for the rs1610696 and rs371194629 variants, it is difficult to formulate a reliable hypothesis to explain the biological mechanism that underlies the observed associations. Lately, the predictive value regarding the genetic predisposition to express different levels of HLA-G was explored in different human cancer cell lines with in vitro functional approaches [54], confirming the intermediate producer signature for UTR-2 haplotype [45]. Moreover, the influence of specific endogenous factors (i.e., RNA-binding proteins and microRNAs), characterizing each cellular type with a particular *HLA-G* 3′UTR haplotype, was highlighted and is currently under investigation by the authors [52]. Recent evidence from both basic and clinical studies has suggested that, in addition to the classical mechanism of drugs binding covalently to DNA, the mechanism of action of platinum-based (including OXA) and FL drugs [26,27,28] could involve immunogenic processes. In recent years, intense interest has driven researchers to investigate the immune system’s ability to recognize and then destroy cancer cells [55]. This study demonstrated that the *HLA-G* 3′UTR SNPs, rs1610696, rs371194629, and the UTR-2 haplotype, should be considered markers with potential clinical value in predicting G3-4 CT-toxicities, particularly toxicities related to neuropathy.

In light of evidence from recent studies that HLA-G is a potent regulator of host immune defenses in patients with cancer and its impact in CRC survival [46,49], we hypothesized that HLA-G might confer tolerance to the inflammatory/immune status induced by drug treatments in CRC. Furthermore, given that platinum-based drugs were associated with enhanced immune system activation, we could not exclude the possibility that HLA-G might play a biological role in the modulation of drug-induced toxicities. However, there is currently a lack of robust data about the functional and biological roles of the *HLA-G* markers analyzed. Therefore, it is difficult to formulate a more detailed hypothesis to explain the mechanisms that might underlie the observed phenotypic effects.

Several life-threatening complications are associated with CT, particularly neurotoxicity, which represents the main dose-limiting toxicity. These complications could have a major impact on the quality of life of patients. Moreover, the adverse reactions associated with FLs/OXA regimens could lead to a dose reduction, treatment delay, and/or therapy suspension, which might potentially compromise the efficacy of FLs/OXA-based therapy and patient benefit. In the context of the immunomodulatory role of HLA-G as an immune checkpoint molecule in cancer [51] and the complex regulation of soluble HLA-G protein expression related to *HLA-G* 3′UTR SNPs and haplotypes [54], our findings represent new advances in the understanding of *HLA-G*.

This study had some limitations. Despite the internal validation (bootstrap analysis), which confirmed some results (particularly rs1610696, rs37119462, and UTR-2), we lacked an independent external validation cohort. Other limitations were the small sample size (*n* = 144) and the retrospective nature of the study. Nonetheless, our analyses resulted in the novel discovery that the *HLA-G* 3′UTR polymorphisms hold value as predictive markers of G3-4 toxicities. Our findings could shed light when exploring the immunogenetic profiles of patients for personalized treatments focused on CRC [56]. Future functional experiments will be required to explore the biological significance of these *HLA-G* 3′UTR markers, once validated, and their involvement in CT-related toxicity. Thus, these preliminary results might have important clinical implications in the management of CRC treatment with adjuvant FLs/OXA-based therapy. Further research is warranted to find novel predictive genetic biomarkers that could be used in clinical practice to reduce morbidity and mortality resulting from CT toxicity.

## 4. Patients and Methods

### 4.1. Patient Characteristics, Study Design, and Toxicity Evaluation

This retrospective study included data from 144 patients with stage II–III CRC that underwent resections with curative intent. The data were retrieved from previous prospective pharmacogenetic [25] and retrospective immunogenetic [49] multicenter studies. Patient eligibility criteria were: histopathologically confirmed stage II–III CRC; radiologically-confirmed absence of distant metastasis; age >18 years; performance status (WHO) 0–2; normal bone marrow, renal, and liver function; and Caucasian ethnicity. Patients were enrolled in centers located in northern and central Italy. Written informed consent was obtained from all participants for the use of blood samples and clinical data for research purposes. The study was approved by the Institutional Review Board of the participating institutes (code CRO-31-2005, 15 September 2005). After diagnosis, eligible patients underwent primary surgery and were treated with a FOLFOX4 regimen, as previously described [25]. Adjuvant CT was continued until completion of the planned cycles, recurrence, toxicity, or patient refusal. The primary endpoint of this study was cumulative G3-4 toxicity (hematological, non-hematological, neutropenia, and neurotoxicity), developed during FOLFOX4 therapy. For each toxicity endpoint, we evaluated the highest grade of toxicity recorded during the treatment to determine associations with *HLA-G* polymorphisms and haplotypes. Toxicity was evaluated according to NCI-CTC criteria version 2.0 (http://ctep.cancer.gov/), as previously described [25]. Briefly, objective clinical evaluations, blood counts, and hepatic and renal function tests were performed within 48 h prior to each cycle. Patients were questioned about nausea and vomiting, mucositis, diarrhea, asthenia (i.e., fatigue, malaise, and weakness), and appetite at the end of every cycle. Neurotoxicity was evaluated according to the OXA-specific scale [25]. Chemotherapy was delayed until recovery from hematological toxicities or any significant, persisting, non-hematological toxicity. In the event of G3-4 toxicity, the doses of OXA and 5-FU were reduced by 25 or 50%, based on the physician’s evaluation. Treatment was discontinued in the event of an anaphylactic reaction, repeated G3-4 toxicity despite a dose reduction, or patient refusal. Due to revisions of the clinical data collected, there are slight differences between the data used in this study and those previously published [25].

### 4.2. HLA-G 3′UTR Polymorphisms and Genotyping Assay

We retrieved germline data collected in a previous study [49] from this cohort of patients with stage II–III CRC. That data described nine *HLA-G* 3′UTR SNPs (Table S2), as follows: rs371194629 (+2960 14-bp INDEL or +2960 14-bp Del/Ins), rs1707 (+3003 T>C), rs1710 (+3010 C>G), rs17179101 (+3027 C>A), rs17179108 (+3035 C>T), rs1063320 (+3142 G>C), rs9380142 (+3187 A>G), rs1610696 (+3196 C>G), and rs123331 (+3227 G>A). Briefly, genomic DNA from peripheral blood was extracted by using the EZ1 DNA Blood Workstation (QIAGEN Inc., Valencia, CA, USA). The 3′UTR of the *HLA-G* gene was amplified by polymerase chain reaction (PCR) using the already published [41] primers HLAG8F: 5′-TGTGAAACAGCTGCCCTGTGT-3′ and HLAG8R: 5′-GTCTTCCATTTATTTTGTCTCT-3′. PCR reactions were carried out in a final volume of 30 μL containing 1.25 mM MgCl2, 0.25 mM of each dNTPs, 5 pmol of each primer, about 50–200 ng of genomic DNA template, 1× PCR Buffer and 0.5 units of AmpliTaq Gold DNA polymerase (Applied Biosystems, Foster City, CA, USA). The PCR cycles were as follows: 5 min of initial denaturation at 94 °C, 30 cycles of 45 s at 95 °C, 45 s at 56 °C, 60 s at 72 °C, and the final extension step at 72 °C for 7 min. Purified reactions (1–2 μL) were sequenced (Sanger method) by the use of the Big Dye Terminator kit (Applied Biosystems, Foster City, CA, USA) and an ABI PRISM capillary sequencer with the reverse HLAG8R primer to prevent sequence overlaps in heterozygous 14-bp samples [49]. Chromatograms were visualized with Chromas software version 2.01 (Technelysium Pty Ltd, South Brisbane, Australia) and all SNPs detected were recorded for each study participant.

### 4.3. Statistical Analyses

This study was designed to test the association between genetic polymorphisms and a wide spectrum of toxicities, namely G3-4 neutropenia and hematological, non-hematological, and neurological toxicities. Odds ratios and 95% confidence intervals (CI) were computed with logistic regression modeling. Models for neutropenia and hematological toxic effects were adjusted for patient age and sex and treatment with neo-adjuvant therapy (yes/no). The estimates for neurological toxic effects were also adjusted for the cumulative OXA dose, normalized by body surface area (mg/m^2^). Dominant, recessive, and additive genetic models were considered for each genotype, including heterozygous and homozygous genotypes. The most statistically significant genetic model was selected according to the Wald *χ*^2^ test. The significance level was set at *p* < 0.05 (two-sided). Results were internally validated with a bootstrap analysis, where the total number of re-samplings was fixed to 1000. Logistic regression analyses were performed with SAS software (version 9.4, SAS Institute, Cary, NC, USA). The R statistical package (version 3.3.1, R Foundation for Statistical Computing, Vienna, Austria) was used for bootstrap internal validations.

## Figures and Tables

**Table 1:** Eligible patient characteristics (*n* = 144).

Variable	*n* (%)
Median age, years (range)	61 (24–82)
Male	82 (56.9)
Female	62 (43.1)
Primary tumor site	
Colon	111 (77.1)
Right	37 (25.7)
Left	69 (47.9)
Transverse/Sigma	5 (3.5)
Rectum	33 (22.9)
Stage at diagnosis	
II	21 (14.6)
III	123 (85.4)
Neo-adjuvant RT (only for rectum)	
Yes	19 (57.6)
No	14 (42.4)
Median number of CT cycles (range)	10.3 (1–12)
Patients that received all 12 planned CT cycles	79 (54.9)
Total (mg) oxaliplatin dose	
Median (range)	1455 (145–2166)
Dose per m^2^ (mg/m^2^): median (range)	819 (84–1050)

RT: radiotherapy; CT: chemotherapy.

**Table 2:** Common cumulative toxicities reported in patients after folinic acid/5-fluorouracil/oxaliplatin (FOLFOX4) treatment.

Toxicity Type	Patients with G3-4 Events (*n* = 144)	
	*n*	%
Hematological	55	38.2
Anemia	0	0.0
Fever with severe neutropenia	2	1.4
Leucopenia	7	4.9
Neutropenia	53	36.8
Thrombocytopenia	1	0.7
Non-Hematological	27	18.8
Alopecia	0	0.0
Asthenia	0	0.0
Cardiac	1	0.7
Constipation	1	0.7
Diarrhea	16	11.1
Hepatic (hyperbilirubinemia)	1	0.7
Infection without severe neutropenia	0	0.0
Mucositis	2	1.4
Nausea	3	2.1
Vomiting	5	3.5
Neurotoxicity	10	6.9

**Table 3:** Associations between *HLA-G* 3′UTR SNPs and G3-4 hematological toxicity.

*HLA-G* 3′UTR SNP	SNP ID	Alleles	Genotypes	*n*	Most Significant Genetic Model			Bootstrap
					Model	OR (95% CI) ^a^	*p*	*p*
+14-bp INDEL	rs371194629	Del	Del/Del	14	Rec	1.69 (0.75–3.81)	0.202	
		Ins	Del/Ins	26				
			Ins/ins	15				
+3003 T>C	rs1707	T	T/T	50	Dom	0.35 (0.12–0.98)	0.046	0.067
		C	T/C	4				
			C/C	1				
+3010 C>G	rs1710	C	C/C	23	Dom	0.71 (0.35–1.43)	0.334	
		G	C/G	26				
			G/G	6				
+3027 C>A	rs17179101	C	C/C	48	Dom	1.36 (0.46–4.04)	0.575	
		A	C/A	7				
+3035 C>T	rs17179108	C	C/C	43	Dom	1.18 (0.49–2.83)	0.707	
		T	C/T	12				
+3142 G>C	rs1063320	G	G/G	23	Dom	0.71 (0.35–1.43)	0.334	
		C	G/C	26				
			C/C	6				
+3187 A>G	rs9380142	A	A/A	31	Rec	0.50 (0.09–2.60)	0.406	
		G	A/G	22				
			G/G	2				
+3196 C>G	rs1610696	C	C/C	22	Rec	3.34 (1.16–9.59)	0.025	0.038
		G	C/G	22				
			G/G	11				

Significant associations are reported in bold (*p* < 0.05); ^a^ Estimated with a logistic regression model adjusted for sex, age, and neo-adjuvant therapy. *HLA-G: human leukocyte antigen-G*; 3′UTR: 3′ untranslated region; SNP: Single nucleotide polymorphism; OR: Odds ratio; CI: Confidence Interval; Rec: Recessive; Dom: Dominant.

**Table 4:** Associations between *HLA-G* 3′UTR SNPs and G3-4 neutropenia toxicity.

*HLA-G* 3′UTR SNP	SNP ID	Alleles	Genotypes	*n*	Most Significant Genetic Model			Bootstrap
					Model	OR (95% CI) ^a^	*p*	*p*
+14-bp INDEL	rs371194629	Del	Del/Del	13	Rec	1.81 (0.80–4.09)	0.154	
		Ins	Del/Ins	25				
			Ins/ins	15				
+3003 T>C	rs1707	T	T/T	48	Dom	0.37 (0.13–1.08)	0.068	
		C	T/C	4				
			C/C	1				
+3010 C>G	rs1710	C	C/C	22	Dom	0.73 (0.36–1.48)	0.389	
		G	C/G	25				
			G/G	6				
+3027 C>A	rs17179101	C	C/C	46	Dom	1.46 (0.49–4.33)	0.497	
		A	C/A	7				
+3035 C>T	rs17179108	C	C/C	41	Dom	1.22 (0.51–2.95)	0.655	
		T	C/T	12				
+3142 G>C	rs1063320	G	G/G	22	Dom	0.73 (0.36–1.49)	0.386	
		C	G/C	25				
			C/C	6				
+3187 A>G	rs9380142	A	A/A	30	Rec	0.51 (0.10–2.67)	0.423	
		G	A/G	21				
			G/G	2				
+3196 C>G	rs1610696	C	C/C	21	Rec	3.76 (1.29–10.96)	0.015	0.042
		G	C/G	21				
			G/G	11				

Significant associations are reported in bold (*p* < 0.05); ^a^ Estimated with a logistic regression model adjusted for sex, age, and neo-adjuvant therapy. *HLA-G: human leukocyte antigen-G*; 3′UTR: 3′ untranslated region; SNP: Single nucleotide polymorphism; OR: Odds ratio; CI: Confidence Interval; Rec: Recessive; Dom: Dominant.

**Table 5:** Associations between *HLA-G* 3′UTR SNPs and G3-4 neurotoxicity.

*HLA-G* 3′UTR SNP	SNP ID	Alleles	Genotypes	*n*	Most Significant Genetic Model			Bootstrap
					Model	OR (95% CI) ^a^	*p*	*p*
+14-bp INDEL	rs371194629	Del	Del/Del	2	Rec	5.49 (1.21–24.85)	0.027	0.022
		Ins	Del/Ins	3				
			Ins/ins	5				
+3003 T>C	rs1707	T	T/T	10	Add	0.00 (0.00–∞)	0.960	
+3010 C>G	rs1710	C	C/C	5	Dom	0.53 (0.14–2.04)	0.357	
		G	C/G	4				
			G/G	1				
+3027 C>A	rs17179101	C	C/C	10	Add	0.00 (0.00–∞)	0.970	
+3035 C>T	rs17179108	C	C/C	8	Add	0.00 (0.00–∞)	0.985	
		T	C/T	2				
+3142 G>C	rs1063320	G	G/G	5	Dom	0.53 (0.14–2.04)	0.357	
		C	G/C	4				
			C/C	1				
+3187 A>G	rs9380142	A	A/A	5	Add	1.37 (0.48–3.91)	0.556	
		G	A/G	4				
			G/G	1				
+3196 C>G	rs1610696	C	C/C	3	Rec	8.78 (1.50–51.44)	0.016	0.019
		G	C/G	3				
			G/G	4				

Significant associations are reported in bold (*p* < 0.05); ^a^ Estimated with a logistic regression model adjusted for sex, age, and neo-adjuvant therapy. *HLA-G: Human leukocyte antigen-G*; 3′UTR: 3′ untranslated region; SNP: Single nucleotide polymorphism; OR: Odds ratio; CI: Confidence Interval; Rec: Recessive; Add: Additive; Dom: Dominant.

**Table 6:** Significant associations between *HLA-G* 3′UTR haplotypes and G3-4 toxicities.

Toxicity Type	Haplotype	14-bp	+3003	+3010	+3027	+3035	+3142	+3187	+3196	*n*	Significant Genetic Models			Bootstrap
											Model	OR (95%-CI) ^a^	*p*	*p*
Hematological	UTR-2	Ins	T	C	C	C	G	A	G		Rec	3.46 (1.14–11.52)	0.028	0.078
	Het									23				
	Hom									10				
Hematological	UTR-4	Del	C	G	C	C	C	A	C		Dom	0.35 (0.12–0.98)	0.046	0.098
	Het									4				
	Hom									1				
Neutropenia	UTR-2	Ins	T	C	C	C	G	A	G		Rec	3.92 (1.28–12.07)	0.017	0.059
	Het									22				
	Hom									10				
Neutropenia	UTR-6	Del	T	G	C	C	C	A	C		Dom	4.77 (1.07–21.20)	0.040	0.058
	Het									6				
	Hom									0				
Neurotoxicity	UTR-2	Ins	T	C	C	C	G	A	G		Rec	11.29 (1.84–69.40)	0.009	0.019
	Het									3				
	Hom									4				

Significant associations are reported in bold (*p* < 0.05); ^a^ Estimated with a logistic regression model adjusted for sex, age, neo-adjuvant therapy, and the oxaliplatin cumulative dose, normalized with body surface area (BSA), for neurological toxicity. *HLA-G: Human leukocyte antigen-G*; 3′UTR: 3′ Untranslated region; OR: Odds ratio; CI: Confidence interval; Het: Heterozygous; Hom: Homozygous; Rec: Recessive; Dom: Dominant.

## Supplementary Materials

Supplementary materials can be found at www.mdpi.com/1422-0067/18/7/1366/s1.

## References

[1] Ferlay J., Soerjomataram I., Dikshit R., Eser S., Mathers C., Rebelo M., Parkin D.M., Forman D., Bray F. (2015). Cancer incidence and mortality worldwide: Sources, methods and major patterns in GLOBOCAN 2012: Globocan 2012. Int. J. Cancer.

[2] Ferlay J., Steliarova-Foucher E., Lortet-Tieulent J., Rosso S., Coebergh J.W.W., Comber H., Forman D., Bray F. (2013). Cancer incidence and mortality patterns in Europe: Estimates for 40 countries in 2012. Eur. J. Cancer.

[3] Price T.J., Segelov E., Burge M., Haller D.G., Ackland S.P., Tebbutt N.C., Karapetis C.S., Pavlakis N., Sobrero A.F., Cunningham D., Shapiro J.D. (2013). Current opinion on optimal treatment for colorectal cancer. Expert Rev. Anticancer Ther..

[4] Cersosimo R.J. (2013). Management of advanced colorectal cancer, part 1. Am. J. Health. Syst. Pharm..

[5] Cersosimo R.J. (2013). Management of advanced colorectal cancer, part 2. Am. J. Health. Syst. Pharm..

[6] Horvat M., Potočnik U., Repnik K., Kavalar R., Štabuc B. (2016). Single Nucleotide Polymorphisms as Prognostic and Predictive Factors of Adjuvant Chemotherapy in Colorectal Cancer of Stages I and II. Gastroenterol. Res. Pract..

[7] DeSantis C.E., Lin C.C., Mariotto A.B., Siegel R.L., Stein K.D., Kramer J.L., Alteri R., Robbins A.S., Jemal A. (2014). Cancer treatment and survivorship statistics, 2014: Cancer Treatment and Survivorship Statistics, 2014. CA. Cancer J. Clin..

[8] Gustavsson B., Carlsson G., Machover D., Petrelli N., Roth A., Schmoll H.-J., Tveit K.-M., Gibson F. (2015). A Review of the Evolution of Systemic Chemotherapy in the Management of Colorectal Cancer. Clin. Colorectal Cancer.

[9] Van Loon K., Venook A.P. (2011). Adjuvant treatment of colon cancer: What is next?. Curr. Opin. Oncol..

[10] Andre T., Boni C., Navarro M., Tabernero J., Hickish T., Topham C., Bonetti A., Clingan P., Bridgewater J., Rivera F., de Gramont A. (2009). Improved Overall Survival with Oxaliplatin, Fluorouracil, and Leucovorin as Adjuvant Treatment in Stage II or III Colon Cancer in the MOSAIC Trial. J. Clin. Oncol..

[11] Argyriou C., Georgiadis G.S., Georgakarakos E.I., Roumeliotis S., Roumeliotis A., Kikas P., Tziakas D., Lazarides M.K. (2015). Applying Evidence-Based Medicine in Actual Clinical Practice: Can We Bridge the Gap? A Review of the Literature. Hell. J. Cardiol..

[12] Brewer J.R., Morrison G., Dolan M.E., Fleming G.F. (2016). Chemotherapy-induced peripheral neuropathy: Current status and progress. Gynecol. Oncol..

[13] Sugihara K., Ohtsu A., Shimada Y., Mizunuma N., Lee P.-H., de Gramont A., Goldberg R.M., Rothenberg M.L., André T., Brienza S. (2012). Safety Analysis of FOLFOX4 Treatment in Colorectal Cancer Patients: A Comparison Between Two Asian Studies and Four Western Studies. Clin. Colorectal Cancer.

[14] Kidwell K.M., Yothers G., Ganz P.A., Land S.R., Ko C.Y., Cecchini R.S., Kopec J.A., Wolmark N. (2012). Long-term neurotoxicity effects of oxaliplatin added to fluorouracil and leucovorin as adjuvant therapy for colon cancer: Results from National Surgical Adjuvant Breast and Bowel Project trials C-07 and LTS-01. Cancer.

[15] Cavaletti G., Alberti P., Marmiroli P. (2011). Chemotherapy-induced peripheral neurotoxicity in the era of pharmacogenomics. Lancet Oncol..

[16] Cortejoso L., López-Fernández L.A. (2012). Pharmacogenetic markers of toxicity for chemotherapy in colorectal cancer patients. Pharmacogenomics.

[17] Fernandez-Rozadilla C., Cazier J.B., Moreno V., Crous-Bou M., Guinó E., Durán G., Lamas M.J., López R., Candamio S., Gallardo E. (2013). Pharmacogenomics in colorectal cancer: A genome-wide association study to predict toxicity after 5-fluorouracil or FOLFOX administration. Pharmacogenom. J..

[18] Ruzzo A., Graziano F., Galli F., Giacomini E., Floriani I., Galli F., Rulli E., Lonardi S., Ronzoni M., Massidda B. (2014). Genetic markers for toxicity of adjuvant oxaliplatin and fluoropyrimidines in the phase III TOSCA trial in high-risk colon cancer patients. Sci. Rep..

[19] Won H.-H., Lee J., Park J.O., Park Y.S., Lim H.Y., Kang W.K., Kim J.-W., Lee S.-Y., Park S.H. (2012). Polymorphic markers associated with severe oxaliplatin-induced, chronic peripheral neuropathy in colon cancer patients. Cancer.

[20] Terrazzino S., Argyriou A.A., Cargnin S., Antonacopoulou A.G., Briani C., Bruna J., Velasco R., Alberti P., Campagnolo M., Lonardi S. (2015). Genetic determinants of chronic oxaliplatin-induced peripheral neurotoxicity: A genome-wide study replication and meta-analysis. J. Peripher. Nerv. Syst..

[21] De Mattia E., Cecchin E., Toffoli G. (2015). Pharmacogenomics of intrinsic and acquired pharmacoresistance in colorectal cancer: Toward targeted personalized therapy. Drug Resist. Updat..

[22] Campbell J.M., Bateman E., Peters M.D., Bowen J.M., Keefe D.M., Stephenson M.D. (2016). Fluoropyrimidine and platinum toxicity pharmacogenetics: An umbrella review of systematic reviews and meta-analyses. Pharmacogenomics.

[23] Toffoli G., Giodini L., Buonadonna A., Berretta M., De Paoli A., Scalone S., Miolo G., Mini E., Nobili S., Lonardi S. (2015). Clinical validity of a DPYD-based pharmacogenetic test to predict severe toxicity to fluoropyrimidines. Int. J. Cancer.

[24] Etienne-Grimaldi M.-C., Renée N., Izzedine H., Milano G. (2009). A routine feasible HPLC analysis for the anti-angiogenic tyrosine kinase inhibitor, sunitinib, and its main metabolite, SU12662, in plasma. J. Chromatogr. B.

[25] Cecchin E., D’Andrea M., Lonardi S., Zanusso C., Pella N., Errante D., De Mattia E., Polesel J., Innocenti F., Toffoli G. (2013). A prospective validation pharmacogenomic study in the adjuvant setting of colorectal cancer patients treated with the 5-fluorouracil/leucovorin/oxaliplatin (FOLFOX4) regimen. Pharmacogenomics J..

[26] Hato S.V., Khong A., de Vries I.J.M., Lesterhuis W.J. (2014). Molecular Pathways: The immunogenic effects of platinum-based chemotherapeutics. Clin. Cancer Res..

[27] De Biasi A.R., Villena-Vargas J., Adusumilli P.S. (2014). Cisplatin-induced antitumor immunomodulation: A review of preclinical and clinical evidence. Clin. Cancer Res..

[28] Lee C.S. (2014). Gastro-intestinal toxicity of chemotherapeutics in colorectal cancer: The role of inflammation. World J. Gastroenterol..

[29] Carosella E.D., Favier B., Rouas-Freiss N., Moreau P., LeMaoult J. (2008). Beyond the increasing complexity of the immunomodulatory HLA-G molecule. Blood.

[30] Curigliano G., Criscitiello C., Gelao L., Goldhirsch A. (2013). Molecular pathways: Human leukocyte antigen G (HLA-G). Clin. Cancer Res..

[31] Amodio G., Sales de Albuquerque R., Gregori S. (2014). New insights into HLA-G mediated tolerance. Tissue Antigens.

[32] Donadi E.A., Castelli E.C., Arnaiz-Villena A., Roger M., Rey D., Moreau P. (2011). Implications of the polymorphism of HLA-G on its function, regulation, evolution and disease association. Cell. Mol. Life Sci..

[33] Garziera M., Toffoli G. (2014). Inhibition of host immune response in colorectal cancer: Human leukocyte antigen-G and beyond. World J. Gastroenterol..

[34] Rouas-Freiss N. (2005). HLA-G Proteins in cancer: Do they provide tumor cells with an escape mechanism?. Cancer Res..

[35] Ye S., Yang H., Li K., Dong D., Lin X., Yie S. (2007). Human leukocyte antigen G expression: As a significant prognostic indicator for patients with colorectal cancer. Mod. Pathol..

[36] Swets M., Seneby L., Boot A., van Wezel T., Gelderblom H., van de Velde C.J.H., van den Elsen P.J., Kuppen P.J.K. (2016). Promoter methylation and mRNA expression of HLA-G in relation to HLA-G protein expression in colorectal cancer. Hum. Immunol..

[37] Guo Z.-Y., Lv Y.-G., Wang L., Shi S.-J., Yang F., Zheng G.-X., Wen W.-H., Yang A.-G. (2015). Predictive value of HLA-G and HLA-E in the prognosis of colorectal cancer patients. Cell Immunol..

[38] Zeestraten E.C.M., Reimers M.S., Saadatmand S., Dekker J.-W.T., Liefers G.J., van den Elsen P.J., van de Velde C.J.H., Kuppen P.J.K. (2014). Combined analysis of HLA class I, HLA-E and HLA-G predicts prognosis in colon cancer patients. Br. J. Cancer.

[39] Zhu C.-B., Wang C.-X., Zhang X., Zhang J., Li W. (2011). Serum sHLA-G levels: A useful indicator in distinguishing colorectal cancer from benign colorectal diseases. Int. J. Cancer.

[40] Cao M., Yie S.-M., Liu J., Ye S.R., Xia D., Gao E. (2011). Plasma soluble HLA-G is a potential biomarker for diagnosis of colorectal, gastric, esophageal and lung cancer. Tissue Antigens.

[41] Castelli E.C., Mendes-Junior C.T., Deghaide N.H.S., de Albuquerque R.S., Muniz Y.C.N., Simões R.T., Carosella E.D., Moreau P., Donadi E.A. (2010). The genetic structure of 3′untranslated region of the *HLA-G* gene: Polymorphisms and haplotypes. Genes Immun..

[42] Castelli E.C., Veiga-Castelli L.C., Yaghi L., Moreau P., Donadi E.A. (2014). Transcriptional and Posttranscriptional Regulations of the *HLA-G* Gene. J. Immunol. Res..

[43] Hviid T.V.F., Hylenius S., Rørbye C., Nielsen L.G. (2003). *HLA-G* allelic variants are associated with differences in the HLA-G mRNA isoform profile and *HLA-G* mRNA levels. Immunogenetics.

[44] Larsen M.H., Hviid T.V.F. (2009). Human leukocyte antigen-G polymorphism in relation to expression, function, and disease. Hum. Immunol..

[45] Martelli-Palomino G., Pancotto J.A., Muniz Y.C., Mendes-Junior C.T., Castelli E.C., Massaro J.D., Krawice-Radanne I., Poras I., Rebmann V., Carosella E.D., Rouas-Freiss N., Moreau P., Donadi E.A. (2013). Polymorphic sites at the 3′ untranslated region of the *HLA-G* gene are associated with differential hla-g soluble levels in the Brazilian and French Population. PLoS ONE.

[46] Garziera M., Catamo E., Crovella S., Montico M., Cecchin E., Lonardi S., Mini E., Nobili S., Romanato L., Toffoli G. (2016). Association of the *HLA-G* 3′UTR polymorphisms with colorectal cancer in Italy: A first insight. Int. J. Immunogenet..

[47] Jeong S., Park S., Park B.-W., Park Y., Kwon O.-J., Kim H.-S. (2014). Human leukocyte antigen-G (HLA-G) polymorphism and expression in breast cancer patients. PLoS ONE.

[48] Ferguson R., Ramanakumar A.V., Koushik A., Coutlée F., Franco E., Roger M., the Biomarkers of Cervical Cancer Risk Study Team (2012). Human leukocyte antigen G polymorphism is associated with an increased risk of invasive cancer of the uterine cervix. Int. J. Cancer.

[49] Garziera M., Bidoli E., Cecchin E., Mini E., Nobili S., Lonardi S., Buonadonna A., Errante D., Pella N., D’Andrea M. (2015). *HLA-G* 3′UTR Polymorphisms Impact the Prognosis of Stage II-III CRC Patients in Fluoropyrimidine-Based Treatment. PLoS ONE.

[50] Sabbagh A., Luisi P., Castelli E.C., Gineau L., Courtin D., Milet J., Massaro J.D., Laayouni H., Moreau P., Donadi E.A., Garcia A. (2014). Worldwide genetic variation at the 3′ untranslated region of the *HLA-G* gene: Balancing selection influencing genetic diversity. Genes Immun..

[51] Carosella E.D., Rouas-Freiss N., Tronik-Le Roux D., Moreau P., LeMaoult J. (2015). HLA-G: An Immune Checkpoint Molecule. Adv. Immunol..

[52] Rizzo R., Audrito V., Vacca P., Brusa D., Stignani M., Bortolotti D., D’Arena G., Coscia M., Laurenti L., Forconi F. (2015). HLA-G is a component of the chronic lymphocytic leukemia escape repertoire to generate immune suppression: Impact of the *HLA-G* 14 base pair (rs66554220) polymorphism. Haematologica.

[53] Bielska M., Bojo M., Klimkiewicz-Wojciechowska G., Jesionek-Kupnicka D., Borowiec M., Kalinka-Warzocha E., Prochorec-Sobieszek M., Robak T., Warzocha K., Mlynarski W. (2015). Human leukocyte antigen-G polymorphisms influence the clinical outcome in diffuse large B-cell lymphoma. Genes Chromosomes Cancer.

[54] Poras I., Yaghi L., Martelli-Palomino G., Mendes-Junior C.T., Muniz Y.C.N., Cagnin N.F., Sgorla de Almeida B., Castelli E.C., Carosella E.D., Donadi E.A. (2017). Haplotypes of the *HLA-G* 3′ Untranslated Region Respond to Endogenous Factors of HLA-G+ and HLA-G- Cell Lines Differentially. PLoS ONE.

[55] Wang J., Reiss K.A., Khatri R., Jaffee E., Laheru D. (2015). Immune Therapy in GI malignancies: A review. J. Clin. Oncol..

[56] Sun X., Suo J., Yan J. (2016). Immunotherapy in human colorectal cancer: Challenges and prospective. World J. Gastroenterol..

