# Supplementary material for: HLA-G 3′UTR Polymorphisms Predict Drug-Induced G3-4 Toxicity Related to Folinic Acid/5-Fluorouracil/Oxaliplatin (FOLFOX4) Chemotherapy in Non-Metastatic Colorectal Cancer
Source: Int J Mol Sci. 2017 Jun 27;18(7):1366. doi: 10.3390/ijms18071366 (PMC5535859; doi:10.3390/ijms18071366)
Supplement: Supplementary file 1 [file ijms-18-01366-s001.pdf]

Supplementary Material: *HLA-G* polymorphisms predict drug-induced G3-4 toxicity related to FOLFOX chemotherapy in non-metastatic CRC

Marica Garziera[1,*], Saverio Virdone [2], Elena De Mattia [1], Lucia Scarabel [1], Erika Cecchin [1], Jerry Polesel[2], Mario D'Andrea [3], Nicoletta Pella [4], Angela Buonadonna [5], Adolfo Favaretto [6], Giuseppe Toffoli[1]

Table S1. Haplotype numbers and frequencies observed at *HLA-G* 3'UTR polymorphic sites in 144 eligible patients with stage II-III CRC

HLA-G 3'UTR Haplotypes[a]	CRC (2*n*=288)	Frequency
UTR-1 DelTGCCCGC	72	0.250
UTR-2 InsTCCCGAG	103	0.358
UTR-3 DelTCCCGAC	41	0.142
UTR-4 DelCGCCCAC	27	0.094
UTR-5 InsTCCTGAC	11	0.037
UTR-6/UTR-18 DelTGCCCAC	9	0.031
UTR-7 InsTCATGAC	17	0.059
UTR-10 DelTCCCGAG	1	0.004
UTR-15 InsTCCCGAC	3	0.010
UTR-38 DelTCCCGGC	1	0.004
UTR-new1? DelTCCTGAG	2	0.007
UTR-new2? DelTCCCCAC	1	0.004

[a]*HLA-G* 3'UTR haplotypes were reconstructed with the PHASE method, according to worldwide distributions previously reported [49,50].

Table S2. Allele and genotype frequencies for *HLA-G* 3'UTR polymorphisms in 144 eligible patients with stage II-III CRC

Polymorphisms	*n* (%)	RefSeq[a]	% EUR MAF[b] (Minor allele)
+2960 14-bp INDEL		rs371194629	37 (Ins)
Del	154 (53.5)		
Ins	134 (46.5)		
Del/Del	41 (28.5)		
Del/Ins	72 (50.0)		
Ins/Ins	31 (21.5)		
+3003 T>C		rs1707	15 (C)
T	261 (90.6)		
C	27 (9.4)		
T/T	119 (82.6)		
T/C	23 (16.0)		
C/C	2 (1.4)		
+3010 C>G		rs1710	46 (C)
C	180 (62.5)		
G	108 (37.5)		
C/C	53 (36.8)		
G/C	74 (51.4)		
G/G	17 (11.8)		
+3027 C>A		rs17179101	6 (A)
C	271 (94.1)		
A	17 (5.9)		
C/C	128 (88.9)		
C/A	15 (10.4)		
A/A	1 (0.7)		
+3035 C>T		rs17179108	9 (T)
C	258 (89.6)		
T	30 (10.4)		
C/C	115 (79.9)		
C/T	28 (19.5)		
T/T	1 (0.7)		
+3142 G>C		rs1063320	46 (G)
G	179 (62.2)		
C	109 (37.9)		
G/G	53 (36.8)		
G/C	73 (50.7)		
C/C	18 (12.5)		
+3187 A>G		rs9380142	34 (G)
A	215 (74.7)		
G	73 (25.4)		
A/A	79 (54.9)		
A/G	57 (39.6)		
G/G	8 (5.6)		
+3196 C>G		rs1610696	28 (G)
C	182 (63.2)		
G	106 (36.8)		
C/C	56 (38.9)		
C/G	70 (48.6)		
G/G	18 (12.5)		
+3227 G>A		rs1233331	4 (A)
G	283 (98.3)		
A	5 (1.7)		
G/G	139 (96.5)		
G/A	5 (3.5)		
A/A	0		

MAF: Minor Allele Frequency; [a]Reference Sequence number for each nucleotide variation is reported in common databases (http://www.1000genomes.org/1000-genomes-browsers and http://www.ncbi.nlm.nih.gov/snp); [b]MAF is reported in the following Caucasian EUR (European) sub-populations, updated 31/01/2017: CEU (Utah residents with Northern and Western European ancestry, N=99), FIN (Finnish subjects in Finland, N=99), GBR (British subjects in England and Scotland, N=91), IBS (Iberian subjects in Spain, N=107), and TSI (Toscani subjects in Italy, N=107).
